# The impact of adding community-based distribution of oral contraceptives and condoms to a cluster randomized primary health care intervention in rural Tanzania

**DOI:** 10.1186/s12978-019-0836-0

**Published:** 2019-12-19

**Authors:** Mallory C. Sheff, Elizabeth F. Jackson, Almamy M. Kanté, Asinath Rusibamayila, James F. Phillips

**Affiliations:** 1 Heilbrunn Department of Population and Family Health, Mailman School of Public Health, Columbia University, 60 Haven Avenue, B2-216, New York, NY 10032 USA; 2 Ifakara Health Institute, Mikocheni, Dar-es-Salaam, Tanzania; 3 Department of International Health Division of Global Disease Epidemiology and Control, Institute for International Programs Bloomberg School of Public Health, Johns Hopkins University, Baltimore, MD USA

**Keywords:** Community health worker, Family planning, Community-based distribution, Tanzania, Primary health care, Reproductive health

## Abstract

**Background:**

Efforts to expand access to family planning in rural Africa often focus on the deployment of community health agents (CHAs).

**Methods:**

This paper reports on results of the impact of a randomized cluster trial of CHA deployment on contraceptive uptake among 3078 baseline and 2551 endline women of reproductive age residing in 50 intervention and 51 comparison villages in Tanzania. Qualitative data were collected to broaden understanding of method preference, reasons for choice, and factors that explain non-use.

**Results:**

Regression difference-in-differences results show that doorstep provision of oral contraceptive pills and condoms was associated with a null effect on modern contraceptive uptake [*p* = 0.822; CI 0.857; 1.229]. Discussions suggest that expanding geographic access without efforts to improve spousal and social support, respect preference for injectable contraceptives, and address perceived risk of side-effects offset the benefits of adopting contraceptives provided by community-based services.

**Conclusions:**

The results of this study demonstrate that increasing access to services does not necessarily catalyze contraceptive use as method choice and spousal dynamics are key components of demand for contraception. Findings attest to the importance of strategies that respond to the climate of demand.

**Trial registration:**

Controlled-Trial.com ISRCTN96819844. Retrospectively registered on 29.03.2012.

## Plain English summary

This paper shares results of a project looking at the contribution of Community Health Agents (CHA) on increasing access to and use of contraceptives in rural Tanzania. Quantitative data for this study included interviewing a total of 3078 baseline and 2551 endline women between the ages of 15 and 49; the qualitative data provided context on women’s preferred family planning method, reasons for this choice, and factors that explain non-use. Results of the analysis demonstrated that the CHA had no impact on increasing women’s use of contraceptives in the study areas. The qualitative data further provided information suggesting that expanding geographic access without improving socio-cultural barriers such as spousal and social support, respecting women’s right to choose her preferred method of contraception, and addressing women’s perceived risk of side-effects counterweigh the positive effect of providing contraceptives directly in the community. Overall, this study demonstrates that increasing community access of some contraceptive methods by CHAs does not necessarily help improve contraceptive use, and that addressing socio-cultural barriers is just as important to instigate change.

## Background

Despite several decades of policy commitment and investment in programs, the widespread need to improve family planning (FP) services persists in much of rural Africa (1–3). Throughout the region, unaddressed demand for contraception contributes to unintended pregnancy (4), sustained high fertility (5) and stalled transitions (6, 7). For four decades, most sub-Saharan countries have pursued policies that attempt to solve this problem with strategies that deploy community workers to distribute care at the community level (8–10). In recent years, this policy has involved shifting a range of public health services to community health workers, including the provision of reproductive health services, thus expanding the pool of trained health workers who are extending the reach of primary health care and family planning to areas where coverage is otherwise sparse (11). Task shifting to improve community-based family planning can also address social constraints to service delivery that are associated with restricting services to clinical environments.

Tanzania is a setting where family planning service reform is urgently needed. Unmet need has remained between 22 and 24% since 1999 despite ubiquitous clinical service points where care is available at no cost, and only half of demand for family planning (53%) is satisfied (12). The widespread persistence of non-use is the consequence of a series of interconnected factors that include fears of method side effects (13), shortages of commodities at facilities (14), lack of provider support (15), and social restrictions on the reproductive autonomy of women (16). Misconceptions and fears around contraceptive use are further exacerbated by gender dynamics, which can introduce suspicion and spousal discord (17). Women who are interviewed about non-use also underscore ways in which the social barriers to using family planning services are compounded by the economic costs of taking time to travel to the facility (16).

In response to these challenges, the Tanzanian Ministry of Health Community Development Gender Elderly and Children (MoHCDGEC) launched a health care program in 2007 that sought to improve access to primary health care, including family planning. Known as the Primary Health Care Services Development Program (*Mpango wa Maendeleo wa Afya ya Msingi*, *MMAM*), the program called for a single, officially compensated national cadre of community health agents (CHAs) to promote the social and economic well-being of Tanzanians through the provision of quality primary health care services at the community level (18). In order to operationalize the CHA component of the MMAM policy, the Ifakara Health Institute (IHI), with technical support from Columbia University’s Mailman School of Public Health (MSPH) and in collaboration with the Tanzanian Ministry of Health Community Development Gender Elderly and Children (MoHCDGEC), launched a cluster randomized trial in 2010 known as the *Connect Project* with the goal of testing the demographic impact of deploying paid, professionalized CHAs in three rural Tanzanian districts (19). The current study is an appraisal of this task shifting component on family planning service delivery.

### Project setting

**Fig. 1:**
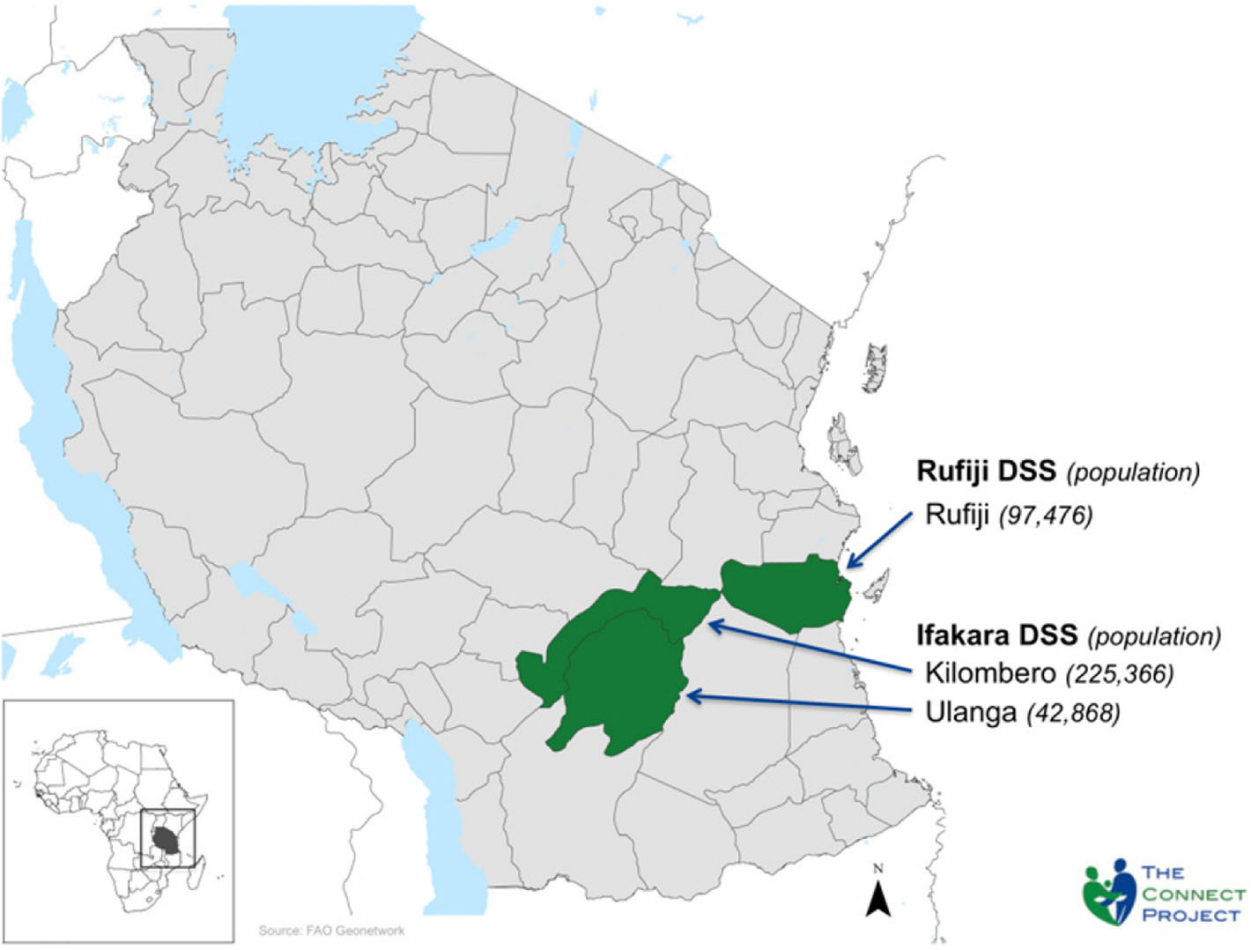
Map of Connect Study area, Rufiji, Kilombero, and Ulanga districts

The Connect Project was implemented in Kilombero, Rufiji, and Ulanga, where the IHI Health and Demographic Surveillance System (HDSS) has monitored population dynamics since 1996 in Kilombero and 1998 in Ulanga and Rufiji (Fig. 1) (20). Kilombero and Ulanga are rural, impoverished districts in the Morogoro Region of Tanzania; Rufiji, also rural, is located in the country’s Pwani Region. At baseline, the population under observation in the project districts was 360,161, 183,367 of which were in the intervention villages. At endline, the population was 379,264, 191,425 of which were in intervention villages. Despite near universal knowledge of one or more contraceptive method, only 34% were using a modern method at the time the Connect Project was launched (21).

### Study design

The Connect Project trial used stratified randomization techniques to allocate 50 villages to the intervention group (villages that received CHAs) and 51 villages to the comparison, with a public drawing to assign villages to each group. The village was chosen as the unit of randomization to align the design of the Connect Project with potential national scale-up of the program as implemented by the Ministry of Health (22). Stratification was segmented into four categories based on village population. Villages in the study area differed substantially by distance to nearest health facility and health facility staffing. Intervention villages received between 1 and 4 CHAs based on population size estimated from the 2009 Demographic Surveillance Survey (23). Overall, 80,300 women were exposed to the Connect Project intervention.

A total of 142 CHAs were trained for 9 months, remunerated, and provided with a comprehensive package of primary health care capabilities, including general health promotion, education and referral for facility care, family planning counseling, distribution of oral contraceptive pills and condoms, safe motherhood promotion, essential newborn care, and essential elements of the WHO Integrated Management of Childhood Illness (IMCI) regimen (24). Council Health Management Teams (CHMT) -- including a Connect Project field coordinator, village authorities, and facility-based health workers-- supervised CHA for their community. Upon deployment, CHA were embedded in local health system structures and became local government employees. They received monthly supportive supervision for their first quarter, and quarterly supervisions afterwards. However, family planning was seen as a component of an integrated MCH service delivery package and was not exclusively monitored.

### Study implementation

CHA postings in the three study districts ran from August 2011 through June 2015. CHA services included household visits and the mobilization of men and women groups for topic-specific interventions, including FP. CHA provided FP counseling to dispel method misconceptions, and distributed condoms and oral contraceptive pills for recurring users at the household level in a manner that would respect the need for privacy and address concerns about access. Workers were instructed to refer first time users and clients who sought other methods to the nearest health center or community dispensary, where depot- medroxyprogesterone acetate (DMPA), intra-uterine devices (IUDs), and implants were available (19). The focus on referral for injectable and long acting methods was mandated by policy; as such, the Connect Project CHA were not trained to provide such services.

This paper examines the impact of shifting tasks of CHA to include family planning in their regimen of care. Given the availability of comprehensive family planning at fixed facilities in Tanzania where their geographic density is intense (25), this study assesses whether there is an incremental effect of doorstep services over and above what is offered at the facility. While dispensary services include the provision of injectables, IUDs, condoms, and oral contraceptive pills, our study examines whether increased community access to contraceptive information and limited methods (pills and condoms) increases use. Using the Connect Project randomized trial as a basis of inference, women in communities randomized to the intervention are hypothesized to have a greater increase of modern contraceptive use over time than women residing in comparison villages.

## Methods

The Connect Project employed a mixed method paradigm involving the conduct of survey research on a random sample of women of reproductive age and a qualitative appraisal of stakeholder reactions to CHA services.

### Quantitative data

were compiled by two household surveys conducted in Swahili in June 2011, to coincide with the onset of community-based care, and July 2015, at the end of the intervention. The same demographic surveillance system clusters were sampled for baseline and endline survey rounds, but household sampling was independent within geographical areas. While individual women of reproductive age (15–49) were interviewed about their demographic, socio-economic, and reproductive health characteristics, including contraceptive use and behavior and pregnancy history, households were the primary sampling unit. A total of 3078 and 2551 women were interviewed on their reproductive health at baseline and endline, respectively.

Independent variables used in the analysis include age, educational attainment, religion, marital status, parity, socio-economic status, and presence or not of a community health agent (CHA). Socioeconomic status was developed using principle component analysis from a series of questions relating to household characteristics such as dwelling unit type, sources of drinking water and type of toilet facility. The outcome variable for the analysis was modern contraceptive use based on the World Health Organization’s list of modern contraceptive methods.

Frequency distributions were compiled with difference-in-differences (DiD) estimators to estimate the effect of CHA on modern contraceptive use in the period between baseline and endline surveys (27). An extended difference in differences procedure applied to the merged baseline and endline data was estimated to refine statistical adjustment for the possible confounding effect of observed covariates while minimizing the confounding effect of unobserved variables that remain constant over time and that are correlated with CHA presence (28). The estimation procedure assumes that exogenous determinants of contraceptive use that could change over the course of the program affect participants in the treatment and control groups equivalently for merged baseline and endline data as given by the linear model:

$$ logit\ {y}_{ij}={\beta}_0+{\beta}_1{time}_i+{\beta}_2{CHA}_i+{\beta}_3\left({time}_i\ast {CHA}_i\right)+{\sum}_{j=1}^J{\gamma}_j{X}_{ij}+\varepsilon $$

where *y* is the log of the odds that individual i with characteristic j, will be a current user of any modern contraceptive method and.

*time* is an indicator of data source for observation i, whereby time = 0 for observations compiled at the baseline and time = 1 for cases observed in the endline survey.

*CHA* is the treatment group dummy for individual i, where CHA = 1 if the household is located in a treatment community and CHA = 0 otherwise.

(*CHA* ∗ *time*) is the DiD indicator of the time by treatment interaction.

*β*_3_ is the difference in differences estimator.

*X*_*ij*_ is the j^th^ control variable for observation i among a vector of J control variables and γ_j_ is the j^th^ parameter of the J control variables.

All statistical analyses were conducted with STATA 12.1.

*Qualitative data* were gathered in the Kilombero district in 2013 in response to evidence from Connect Project midline survey research showing that CHA deployment was not associated with increased contraceptive use, despite community deployment for two years. Interviews from 48 women from 4 focus group discussions (FGDs) and 8 influential community members from in-depth interviews (IDIs) were analyzed. FGDs had an average of 12 individuals, and were interviewed by age group— young women aged 15 to 29 were separated from those aged 30 to 49; 4 men and 4 women were included in the IDIs. Interviewees were recruited by asking village leaders to appoint local focal individuals to help identify individuals for participation.

Qualitative data collection instruments were open-ended discussion guides that focused on determinants and barriers to primary health care service utilization, knowledge of family planning, perceptions of family planning services, contraceptive utilisation, and other contextual factors contributing to these themes. FGD facilitators were research assistants from Tanzania, fluent in Swahili and local dialects. Interviews were conducted in Swahili, recorded, and transcribed the day of the interviews. The data were then translated into English by bilingual translators and transcribed for analysis. Transcripts were analyzed using a thematic analysis approach in which qualitative analysis is completed in six phases, including: 1) familiarization with and organization of the data, 2) generating initial codes, 3) searching for themes, 4) theme review, 5) defining and naming the themes, and 6) producing a report (29).

### Limitations

Multi-level analyses of the interaction of the health system with the social system would clarify social system determinants of family planning behavior. For example, the needs and perspectives of men, and the impact of male preferences on women’s contraceptive behavior, would merit systematic research and appraisal (30–32). Other social determinants of choice and behavior such as socio-cultural norms and societal expectations of behavior would have also warranted further study. The validity of the qualitative research component of this manuscript would have been further strengthened by engaging in group-based coding. One researcher conducted the coding process alone. As a result, inter-rater reliability was not calculable.

## Results

### Population characteristics

Table 1 reports marginal frequency distributions for the descriptive characteristics of women interviewed at baseline and endline by treatment and comparisons villages. Results show that at baseline, the study is generally balanced with the exception of a statistically significant difference in wealth index (*p* < 0.003) and slight difference in religion (*p* < 0.053). As per Table 1, while significant, the effect size difference for the latter is quite small. Overall, the majority of women at baseline are under the age of 30 (53.6 and 52.1% in control and treatment areas, respectively), have completed less than secondary education, and are married. Women living in the poorest households (1st wealth quintile) comprised 22.5 and 27.5% of the respondents in comparison and treatment villages, respectively.

**Table 1:** Descriptive characteristics of women at baseline and endline in treatment and comparison areas

Variable		Baseline (2011)			Endline (2015)		
		Treatment = 0	Treatment = 1	*p*-value	Treatment = 0	Treatment = 1	*p*-value
Age	15–24	592 (38.6%)	595 (38.6%)	0.11	471 (37.1%)	467 (37.3%)	0.82
	25–29	231 (15.0%)	208 (13.5%)		161 (12.7%)	141 (11.3%)	
	30–34	215 (14.0%)	243 (15.8%)		166 (13.1%)	173 (13.8%)	
	35–39	242 (15.8%)	207 (13.4%)		173 (13.6%)	179 (14.3%)	
	40–49	255 (16.6%)	287 (18.6%)		298 (23.5%)	292 (23.3%)	
Education	No education	278 (18.1%)	281 (18.2%)	0.86	237 (18.7%)	211 (16.9%)	0.42
	Less than secondary	1031 (67.2%)	1043 (67.7%)		828 (65.2%)	845 (67.5%)	
	Secondary or more	226 (14.7%)	216 (14.0%)		204 (16.1%)	196 (15.7%)	
Wealth index/ SES Status	Poorest	345 (22.5%)	424 (27.5%)	0.003**	229 (18.0%)	234 (18.7%)	0.003**
	Poor	313 (20.4%)	279 (18.1%)		174 (13.7%)	232 (18.5%)	
	Better	268 (17.5%)	289 (18.8%)		282 (22.2%)	224 (17.9%)	
	Less poor	343 (22.3%)	287 (18.6%)		228 (18.0%)	240 (19.2%)	
	Least poor	266 (17.3%)	261 (16.9%)		295 (23.2%)	276 (22.0%)	
	Missing data	–	–		61 (4.8%)	46 (3.7%)	
Religion	Christian	703 (45.9%)	740 (48.1%)	0.053	559 (44.1%)	593 (47.4%)	0.009**
	Muslim	774 (50.5%)	765 (49.7%)		634 (50.0%)	618 (49.4%)	
	Traditional/ Other	53 (3.5%)	35 (2.3%)		73 (5.8%)	39 (3.1%)	
	No response/ missing				3 (0.2%)	2 (0.2%)	
Parity	0	344 (22.4%)	325 (21.1%)	0.20	349 (27.5%)	346 (27.6%)	0.59
	1–2	419 (27.3%)	439 (28.5%)		345 (27.2%)	312 (24.9%)	
	3–4	394 (25.7%)	358 (23.3%)		282 (22.2%)	288 (23.0%)	
	5+	378 (24.6%)	417 (27.1%)		293 (23.1%)	306 (24.4%)	
Marital status	Married	820 (53.4%)	856 (55.6%)	0.84	597 (47.0%)	620 (49.5%)	0.23
	Living with partner	172 (11.2%)	162 (10.5%)		165 (13.0%)	156 (12.5%)	
	Divorced	87 (5.7%)	87 (5.6%)		77 (6.1%)	94 (7.5%)	
	Widowed	33 (2.1%)	28 (1.8%)		28 (2.2%)	31 (2.5%)	
	Single	421 (27.4%)	406 (26.4%)		400 (31.5%)	347 (27.7%)	
	No response/ Missing	2 (0.1%)	1 (0.1%)		2 (0.2%)	4 (0.3%)	
Modern use of FP		496 (32.3%)	508 (33.0%)	0.69	388 (30.6%)	393 (31.4%)	0.66
Number of respondents		1535	1540		1269	1252	

**p < 0.01

At endline, differences between treatment and control areas are statistically significant with respect to socioeconomic status (*p* = 0.003) and religion (*p* = 0.009). Almost half of women interviewed were under 30 (49.8% in comparison villages and 48.6% in treatment villages) and married (47% in comparison villages and 49.5% in treatment villages).

### Modern contraceptive use

Results in Table 2 present modern contraceptive use by covariate at baseline and endline. Women aged 15–24 have a higher prevalence of modern contraceptive relative to older women with 32% using a modern method at baseline, and 23% at endline. Likewise, women with some education were more frequent users of contraception than women with no education, with 73% of users at baseline and endline; contraceptive use prevalence was comparable by religion.. Married women were more prevalent users of modern methods than unmarried women living with a partner or divorced, widowed, or single women.

Table 3 further details contraceptive use by method type at baseline and endline by treatment and comparison village. The decrease in use of oral contraceptive pills at endline in both the treatment and comparison villages is notable, despite distribution of this method by CHA at the doorstep level. The increased use of implants is furthermore notable with a nearly twofold increase between baseline and endline; there is, however, no significant difference between treatment and control villages. In general, oral contraceptive pills, injectables, and implants are the most used modern contraceptive methods for women in our project area, with injectables the clear contraceptive method of choice.

**Table 3:** Modern contraceptive use at baseline and endline by treatment and control areas

Variable	Baseline (2011)		Endline (2015)	
	Treatment = 0	Treatment = 1	Treatment = 0	Treatment = 1
emale Sterilization	22 (1.42%)	24 (1.68%)	25 (1.97%)	28 (2.24%)
Male Sterilization	1 (0.06%)	4 (0.28%)	–	–
Pill	124 (8.02%)	126 (8.81%)	77 (6.07%)	67 (5.35%)
IUD	16 (1.03%)	17 (1.19%)	18 (1.42%)	17 (1.36%)
Injectables	216 (13.97%)	197 (13.78%)	148 (11.66%)	178 (14.22%)
Implants	38 (2.46%)	28 (1.96%)	58 (4.57%)	46 (3.67%)
Male Condom	63 (4.08%)	63 (4.41%)	51 (4.02%)	46 (3.67%)
Female Condom	5 (0.32%)	5 (0.35%)	1 (0.08%)	1 (0.08%)
Emergency Contraception	1 (0.06%)	0	2 (0.16%)	2 (0.16%)
Lactation Amenorrhea Method	0	1 (0.07%)	4 (0.32%)	2 (0.16%)
Other Modern Method	7 (0.45%)	11 (0.77%)	4 (0.32%)	6 (0.48%)
Total	1546	1430	1269	1252

**Table 2:** Modern contraceptive prevalence by covariate at baseline and endline

Variable		Baseline (2011)			Endline 2015		
		Mod FP = 0	Mod FP = 1	p-value	Mod FP = 0	Mod FP = 1	p-value
Age	15–24	869 (42.0%)	318 (31.7%)	< 0.001*	770 (43.8%)	181 (22.8%)	< 0.001**
	25–29	257 (12.4%)	182 (18.1%)		175 (10.0%)	129 (16.3%)	
	30–34	258 (12.5%)	200 (19.9%)		191 (10.9%)	152 (19.2%)	
	35–39	278 (13.4%)	171 (17.0%)		196 (11.1%)	160 (20.2%)	
	40–49	409 (19.7%)	133 (13.2%)		426 (24.2%)	171 (21.6%)	
Education	No education	418 (20.2%)	141 (14.0%)	< 0.001**	321 (18.3%)	134 (16.9%)	< 0.001**
	Less than secondary	1341 (64.7%)	733 (73.0%)		1112 (63.3%)	579 (73.0%)	
	Secondary or more	315 (15.2%)	130 (12.9%)		325 (18.5%)	80 (10.1%)	
Wealth index/ SES Status	Poorest	543 (26.2%)	226 (22.5%)	<0.032*	312 (17.7%)	153 (19.3%)	< 0.001**
	Poor	395 (19.0%)	197 (19.6%)		288 (16.4%)	127 (16.0%)	
	Better	352 (17.0%)	205 (20.4%)		320 (18.2%)	195 (24.6%)	
	Less poor	438 (21.1%)	192 (19.1%)		356 (20.3%)	121 (15.3%)	
	Least poor	343 (16.5%)	184 (18.3%)		396 (22.5%)	176 (22.2%)	
	Missing data	3 (0.1%)	0 (0.0%)		86 (4.9%)	21 (2.6%)	
Religion	Christian	955 (46.2%)	488 (48.6%)	< 0.001**	783 (44.5%)	380 (47.9%)	< 0.001**
	Muslim	1025 (49.5%)	514 (51.2%)		863 (49.1%)	407 (51.3%)	
	Traditional/ Other	86 (4.2%)	2 (0.2%)		106 (6.0%)	6 (0.8%)	
	No response/ Missing	3 (0.1%)	0 (0.0%)		6 (0.3%)	0 (0.0%)	
Parit**y**	0	565 (27.3%)	107 (10.7%)	< 0.001**	561 (31.9%)	143 (18.0%)	< 0.001**
	1–2	545 (26.3%)	313 (31.2%)		449 (25.5%)	218 (27.5%)	
	3–4	454 (21.9%)	298 (29.7%)		351 (20.0%)	221 (27.9%)	
	5+	509 (24.6%)	286 (28.5%)		397 (22.6%)	211 (26.6%)	
Marital status	Married	1066 (51.5%)	610 (60.8%)	< 0.001**	805 (45.8%)	426 (53.7%)	< 0.001**
	Living with partner	206 (9.9%)	128 (12.7%)		193 (11.0%)	132 (16.6%)	
	Divorced	126 (6.1%)	48 (4.8%)		115 (6.5%)	60 (7.6%)	
	Widowed	46 (2.2%)	15 (1.5%)		49 (2.8%)	10 (1.3%)	
	Single	625 (30.2%)	202 (20.1%)		590 (33.6%)	165 (20.8%)	
	No response/ Missing	2 (0.1%)	1 (0.1%)		6 (0.3%)	0 (0.0%)	
In Treatment areas		1032 (49.8%)	508 (50.6%)	0.69	859 (49.4%)	393 (50.3%)	0.66
Number of Respondents		2074	1004		1758	793	

**p < 0.01

**Table 4:** Regression difference-in-differences (DiD) estimates of the impact of CHAs on modern contraceptive use adjusting for the effects of socio-demographic covariates

Covariates	Odds Ratio	*P* > t	[95% Conf. Interval]	
*DiD (Ref: Baseline)*				
Post (Endline)	1.026	0.779	.857	1.229
*Village (Ref: Control Village)*				
Treatment Village	1.000	0.999	.839	1.191
DiD Interaction (Post*Treatment)	1.026	0.822	.819	1.286
Age	1.456	0.000**	1.387	1.528
Age squared	.994	0.000**	.993	.995
*Education (ref: no education)*				
Less than secondary education	1.438	0.000**	1.202	1.719
Secondary education or more	1.480	0.002**	1.154	1.898
*Religion (Ref: Christian)*				
Muslim	1.000	0.992	.879	1.140
Traditional or other	.072	0.000**	.035	.149
*Marital Status (Ref: Married)*				
Living with partner	1.154	0.159	.946	1.409
Divorced	.739	0.015*	.580	.942
Widowed	.567	0.003**	.388	.828
Single	.847	0.073	.707	1.016
*Wealth Quintile (Ref: Poorest)*				
Poor	1.013	0.909	.806	1.274
Better	1.299	0.004**	1.086	1.553
Less Poor	.940	0.591	.751	1.177
Least Poor	1.101	0.422	.871	1.392
Parity	1.075	0.000**	1.035	1.118
_cons	.002	0.000**	.001	.003

Summary statistics *p < 0.05 **p < 0.01

Observations: 5469

Wald chi2 (18) 522.36

Prob > chi2 0.000

Pseudo R^2^: 0.0718

### Difference in differences estimation

Table 4 presents the DiD model showing baseline-endline differences with period effects capturing temporal change, and the treatment-period interaction representing the DiD. Other rows of Table 4 present nuisance parameters that adjust for age, educational attainment, religion, marital status, socio-economic status, and parity. Results show that the difference-in-difference estimates no effect of the presence of community health workers on modern contraceptive use [*p* = 0.822; CI 0.819; 1.286].

### Qualitative data

Qualitative data were compiled to provide a deeper understanding of community acceptance of CHA, women’s method preference, and reasons for this preference as factors that could explain why CHA did not have an effect on women’s modern contraceptive uptake despite community engagement for four years.

### CHA acceptance in the community

CHA acceptance in intervention communities was overwhelmingly positive, with both CHA and community members reporting good community engagement and exchanges. CHA felt welcomed by community members who did not express fear or discontent with their services.

*Facilitator: To what extent does the community accept your services?*

*Respondent: In fact, the community has received us positively (…*) *they have seen the advantages [of our services]. For example, environmental cleanliness, family planning, and the treatment of children under the age of 5 years. They [the community] respond positively because they have seen the advantages.*

*F: How have they received the services?*

*R: They have received these services positively to a large extent because they are using them and the education that we have given them is applied.*

*F: Have you ever received complaints or fear from the community towards your services?*

*R: In fact, that has never happened.*

As above, women and men reported accepting CHA into their communities. Many were grateful for the health and educational services provided the CHA.

*Respondent: No one fears them [CHAs], everybody is satisfied based on their performance. In fact, these CHAs are trying their level best and they are doing a good job.*

*Facilitator: there is no one who has a fear or worries?*

*P6: No one has any fear or anxiety.*

*In short, we don’t have any fear with CHAs, we accept them and we want them to proceed with their job. We like them.*

Interaction with CHA on family planning education and the provision of condoms and pills was also positively received by community members.

*I interact with CHAs in the issue of family planning, so when I need (something), they come to give me education. They can also provide me with pills or condoms, they can advise me on the methods I want to use, or they can mention all methods and I can choose the preferred one because they help.*

### Contraceptive method preference

Despite overwhelming acceptance of CHA in the community, their limited distribution of condoms and oral contraceptive pills to recurring users did not align with women’s method preference. The favored contraceptive methods cited in the interviews were pills and DMPA, with overall preference for injectables. Indeed, while interviewees often cited both contraceptive methods, apprehension about missing a day offset women’s desire to use oral contraception:

*Others don’t prefer using pills because they worry that they will forget to take the pills daily. So they prefer to take injections because these last for three months.*

*You know, every drug of family planning has it fans, you may find others they tell you ‘I like pills more’ but there are very few. You may also find someone tell you to swallow pills every day is a problem if you travel. Me, I see injection is best because you get injection which works after three months ( … ). Many young girls use injections more.*

In general, women tended to favor family planning methods that could be pursued in secret, away from the husband’s knowledge. In the traditional setting of rural Tanzania, as in gender stratified traditional settings elsewhere in rural sub-Saharan Africa, men attempt to control household fertility decisions:

*Most of the time, I should be honest, the man is the one who makes the decision, and if is not, then he will think something wrong.*

*In my opinion, others have a lot of children because they are not allowed to use family planning pills. [(Facilitator: Who restricts them?] Their partners.*

Injections were thus preferred because their discreet application permits secrecy from their husbands’ knowledge. Unlike pills, which women have to take daily in their homes, injectable contraceptives (DMPA) are done once every three months at the facility:

*They [men] want [child] bearing. So they make women use [contraceptives] in secret. A woman may pretend she is sick, she may go to hospital and the nurse gives her an injection. And injections don’t show any sign that you have gotten it ( … ).*

*If your husband did not understand [about family planning] you will use injection ( … ).*

While women highlighted the importance of surreptitious use of family planning, they also underscored the socio-cultural barriers that foster this concern. Interviewees believed that if family planning promotional and educational activities could focus on couples rather than individual women, such encounters would enhance the social acceptability of family planning use:

*Men should also be given education like this, because some men are so against contraceptives. They say family planning is against God, because God gives you the capability to reproduce, and if you force him to use contraceptives, you will be beaten and you should conceive.*

*We can use CHA at the time when they come. There should be announcements that CHA will be at certain area certain days, so every man and woman should attend. As you know nowadays even men are supposed to attend the clinic so as to be aware what their wives have been told. Other men have a clear understanding, so when they receive such education they agree with family planning methods.*

## Discussion

The aim of this study was to assess the incremental impact of deploying professionally trained community health workers to dispense oral contraceptive pills and condoms at the household level in three rural districts of Tanzania. The strategic design of the project was compliant with official policies that constrain the range of services that community-based workers are allowed to provide. Oral contraceptive pill users were required to initiate use in clinics and dispensaries; CHA, in turn, were restricted to resupplying existing users and informational exchanges with potential new users. DMPA was available in nearby clinics and dispensaries, but nonclinical household and community-based services delivered by CHA did not provide this or other long-acting methods. The operational model of official policy therefore constrained the range of services CHA could provide.

Results demonstrate that the deployment of paid, professional CHA who provided family planning education, oral contraceptive pills, and barrier methods at the doorstep did not engender increased contraceptive use over and above levels of family planning practice that were observed in comparison communities, despite 61% of respondents reporting being visited by a CHA in the last 3 months. Findings bring into question the efficacy of policies that attempt to expand access to family planning without also expanding the range of contraceptive methods. Studies on the provision of injectable family planning services by community workers have been conducted in Ethiopia, Uganda, Kenya, Senegal, Ghana, Malawi, Nigeria and elsewhere (33–35). And, following a technical consultation review, the World Health Organization (WHO), the United States Agency for International Development (USAID), and Family Health International (FHI) concluded that evidence supports the introduction and scale-up of community-based DMPA, with support for its inclusion in policy and operational guidelines in developing countries (36). In countries with rapid contraceptive uptake, this has resulted in a 50 to 75% utilization of injectables as women’s family planning method of choice (37).

Improving method access also involves strategies for improving social access to contraception. In particular, mapping and understanding the social context when designing family planning programs requires strategic planning on how best to involve men in the process of ensuring the social acceptability of contraceptive services and practice (38, 39). Our findings on the importance of gender inclusion are not new: Three decades ago, Easterlin and colleagues posited a framework that specified the importance of mitigating social and spousal constraints to fertility regulation (40, 41). Gender sensitive programming remains important in ensuring a comprehensive approach to social acceptance of family planning utilization (42). Yet, while social access to family planning services is important, social, demographic, and behavioral research in Tanzania and elsewhere has consistently demonstrated the importance of maximizing the range of contraceptive options alongside increased access to care (43, 44). Expanding method options contributes to the goal of improving the quality of care (45, 46), reproductive rights, and social acceptability of contraception (47).

With so much evidence to support policies that expand choice and improve social support for family planning, why is more research on this topic needed? The Connect Project results attest to the incongruence of Tanzania’s restriction on the range of methods that community workers are allowed to provide with the method preferences of the population that such workers serve. A truly comprehensive program would be an integrated approach to primary health care with community-engagement of social networks that addresses the socio-cultural environment (48, 49), including the concerns of men and behavioral expectations, while also providing doorstep access to a range of methods that respects the preferences of women.

## Conclusion

The hypothesis that contraceptive use will increase if access to a limited range of FP services is provided by community-based care is often accepted without consideration of the socio-cultural factors that define the climate of demand. The results of this study demonstrate that increasing access to services does not necessarily catalyze contraceptive use; method choice and spousal dynamics are some of the key components of FP demand. The importance of including gender strategies that respond to the demand for methods that permit covert use is also emphasized. These findings invite review of Tanzania’s operational policies that restrict the role of community workers, with a focus on implementation research conducted elsewhere in Africa and three decades ago in Asia (50–53). Social factors that prevent women from using their family planning method of choice invite policies that offset such constraints with convenient access to the full range of methods that community workers can offer. Studies fielded in East and Southern Africa show that expanding the range of methods provided by community-based services beyond one or two can provide additional benefits to women in need of care, even if these methods are readily available in clinics and dispensaries.

## Data Availability

Africa Health Initiative coordination mechanisms sponsored by the Doris Duke Charitable Foundation supported a Data Cooperative for organizing international access to Africa Health Initiative project data sets. The project protocol and data used for this analysis and data collection instruments are available upon written request to the Data Cooperative by emailing the lead author of this publication, with a copy to the corresponding author. Data of the Connect Project are described at https://www.jhsph.edu/research/centers-and-institutes/institute-for-international-programs/_documents/phit_concept.pdf. Core indicators for this study are displayed at: https://dataverse.harvard.edu/file.xhtml?persistentId=doi:10.7910/DVN/26539/PN40RD.

## Abbreviations

CHA: Community Health Agent
CHMT: Council Health Management Teams
DMPA: Depot- medroxyprogesterone acetate
FGD: Focus group discussions
FHI: Family Health International
FP: Family Planning
HDSS: Health and Demographic Surveillance System
IDI: In-depth interviews
IHI: Ifakara Health Institute
IMCI: Integrated Management of Childhood Illness
IUD: Intra-uterine device
MoHCDGEC: Tanzanian Ministry of Health Community Development Gender Elderly and Children
MSPH: Mailman School of Public Health
USAID: United States Agency for International Development
